# A Novel 3D-Printed and Miniaturized Periodic Counter Current Chromatography System for Continuous Purification of Monoclonal Antibodies

**DOI:** 10.3390/mi15030382

**Published:** 2024-03-13

**Authors:** Carlotta Kortmann, Taieb Habib, Christopher Heuer, Dörte Solle, Janina Bahnemann

**Affiliations:** 1 Institute of Technical Chemistry, Leibniz University Hannover, 30167 Hannover, Germany; kortmann@iftc.uni-hannover.de (C.K.); habib@iftc.uni-hannover.de (T.H.); solle@iftc.uni-hannover.de (D.S.); 2 Chair Technical Biology, Institute of Physics, University of Augsburg, 86159 Augsburg, Germany; christopher.heuer@uni-a.de; 3 Centre for Advanced Analytics and Predictive Sciences (CAAPS), University of Augsburg, 86159 Augsburg, Germany

**Keywords:** 3D-printing, continuous chromatography, miniaturization, monoclonal antibody

## Abstract

Continuous chromatography has emerged as one of the most attractive methods for protein purification. Establishing such systems involves installing several chromatographic units in series to enable continuous separation processes and reduce the cost of the production of expensive proteins and biopharmaceuticals (such as monoclonal antibodies). However, most of the established systems are bulky and plagued by high dead volume, which requires further optimization for improved separation procedures. In this article, we present a miniaturized periodic counter-current chromatography (PCCC) system, which is characterized by substantially reduced dead volume when compared to traditional chromatography setups. The PCCC device was fabricated by 3D printing, allowing for flexible design adjustments and rapid prototyping, and has great potential to be used for the screening of optimized chromatography conditions and protocols. The functionality of the 3D-printed device was demonstrated with respect to the capture and polishing steps during a monoclonal antibody purification process. Furthermore, this novel miniaturized system was successfully used for two different chromatography techniques (affinity and ion-exchange chromatography) and two different types of chromatographic units (columns and membrane adsorbers). This demonstrated versability underscores the flexibility of this kind of system and its potential for utilization in various chromatography applications, such as direct product capture from perfusion cell cultures.

## 1. Introduction

Monoclonal antibodies (mAb) are proteins that specifically recognize antigenic epitopes [1] and thus have great potential for targeted therapeutic uses [2]. For instance, mAbs are used to alleviate autoimmune diseases such as multiple sclerosis [3,4] and rheumatoid arthritis [5,6,7] or for the treatment of cancer [8,9]. mAbs are preferably produced in mammalian cells such as Chinese hamster ovary (CHO) cells, since these cells generate the most human-like glycosylation patterns and thereby support immunogenic safety and efficacy [10]. While the upstream process for mAb production in CHO cells has been intensively studied and well-optimized (e.g., product titer, process costs, quality, etc.) [11,12,13], much less optimization has been achieved in the downstream process (e.g., mAb recovery and purification). This is in large part because the downstream procedure is much more complex in nature. Currently, several different chromatography steps in batch mode are recommended in order to obtain a high-quality mAb product. These steps typically include Protein A-based affinity chromatography, which is applied to the clarified supernatant and captures antibodies by binding the Fc-fragment of immunoglobulin [14,15]. In contrast, other components—such as cell debris or host cell proteins (HCP)—flow through the chromatographic unit (CU) unimpeded [16]. Additional chromatographic steps (e.g., ion exchange and hydrophobic interaction) are typically applied to remove remaining impurities like HCPs, residual nucleic acid, and aggregates [17,18].

Batch chromatography can, however, only keep up with the increasing titers of the upstream process to a limited extent [19]. The primary reason for this inefficiency is that the columns are often loaded only up to 10% product breakthrough (due to the risk of product loss). To alleviate this issue, multi-column approaches within a continuous chromatography system are usually deployed, and the columns are loaded via a continuous or semi-continuous feed stream. This means that the unbound product is directed from column to column, so that previously loaded columns can be replenished. Several different continuous approaches are now available and include simulated moving bed (SMB) chromatography and periodic counter-current chromatography (PCCC) [20,21,22]. For example, in PCCC, the CUs are temporarily loaded up in series to capture the breakthrough of the first unit and thereby achieve higher resin utilization. By using these continuous systems, product loss and buffer consumption can be reduced and space–time yield is improved. All these are essential factors that collectively enhance productivity and reduce the carbon footprint overall compared to batch chromatography [16,21,23,24].

Despite these advantages, however, most systems designed for continuous chromatography are voluminous and can be difficult to both set up and customize. Furthermore, they are also quite complex and require a considerable amount of tubing as well as a multitude of valves [25]. To mitigate these issues, we designed a miniaturized PCCC system in which the tubing is largely replaced by a 3D-printed device which features an integrated mixing unit. This system is an advancement of our previous device, which demonstrated the tremendous potential offered by 3D printing to reduce both the dead volume and the size of batch chromatography significantly [26]. This novel 3D-printed PCCC device also enabled a fully automated and continuous purification process with further improved space–time yield and reduced buffer consumption due to the lower dead volume. Moreover, 3D printing fabrication facilitated rapid prototyping and flexible design adjustments [27,28]—a consideration that has a substantially more pronounced impact on continuous systems. For instance, in a continuous chromatography system, the modular and flexible connection of pumps, chromatographic separation units, detectors, and fraction collectors in varying numbers is crucial. Three-dimensional printing offers unmatched design flexibility and straightforward design fabrication.

In summary, the presented PCCC system holds great potential to be used for the efficient screening of optimized mAb purification processes—especially within bioprocess research-intensive laboratories, where it can potentially be directly coupled to perfusion cell culture (due to the flexibility of 3D printing).

## 2. Materials and Methods

### 2.1. Fabrication of the Miniaturized 3D-Printed PCCC System

**Figure A1:**
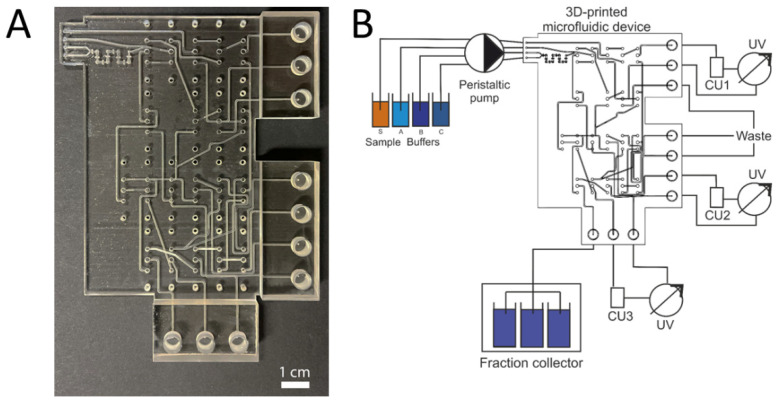
(**A**) Photograph of the 3D-printed PCCC system. A UV-curable lacquer was applied to the device to increase its transparency and make the internal microfluidic channels visible. (**B**) CAD drawing of the 3D-printed PCCC system for continuous chromatography, including the periphery such as buffer reservoirs, fraction collector, chromatographic units (CUs), and UV-photometers (UVs).

**Figure A2:**
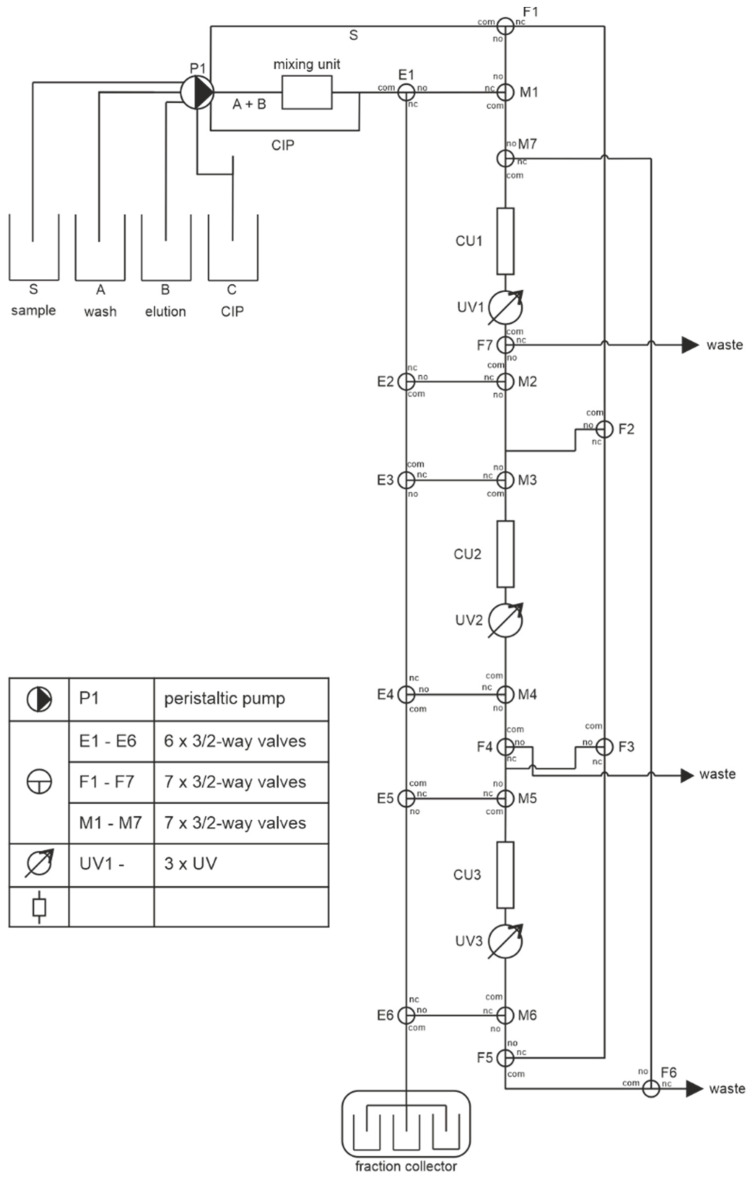
Circuit diagram of the miniaturized PCCC device. P1: peristaltic pump; E1–E6, F1–F7, M1–M7: valves; UV1–UV3: UV photometers; CU1-CU3: chromatography units. A total of 20 magnetic 3/2 solenoid valves are required to enable the fully automated switching of the channels. NO: normally open; NC: normally closed; COM: common.

The miniaturized PCCC device was designed as a CAD model using SolidWorks 2021 (Dassault Systèmes, Vélizy-Villacoublay, France) and then 3D-printed using the high-resolution 3D printer ProJet^®^ MJP 2500 Plus (3D Systems, Rock Hill, SC, USA). A CAD drawing, and the circuit design of the miniaturized PCCC, with further details, are included in Appendix A (Figure A1 and Figure A2). The printer was operated in “ultra high definition” mode, with a nominal resolution of 1600 × 900 × 790 DPI and a layer height of 32 µm [29]. A biocompatible and heat-resistant UV-photocurable acrylate material (VisiJet^®^ M2S-HT90 (3D Systems, Rock Hill, SC, USA)), which has been previously selected for various biotechnology applications, was used as the printing material [28,30]. The support material was a water-soluble hydroxylated wax VisiJet^®^ M2 SUP [31]. After the printing process was completed, the printing plate was incubated for 10 min at −18 °C, in order to facilitate the easy removal of the 3D-printed objects. Subsequently, the 3D-printed PCCC device was incubated in a hot steam bath and a hot paraffin oil (Carl Roth GmbH + Co. KG, Karlsruhe, Germany) bath to remove the wax support material. The residual oil was removed via treatment in a hot ultrasonic water bath with detergent (Fairy Ultra, Procter & Gamble, Cincinnati, OH, USA).

### 2.2. Experimental Setup of the Continuous Miniaturized Chromatography System

Buffers, cell culture supernatant (Protein A), and mAb solution (CEX) as feed solution were stored in respective glass bottles (Schott Duran, Germany) and connected to a 4-channel peristaltic pump (Ismatec REGLO ICC, Cole-Palmer GmbH, Wertheim, Germany) with Tygon^®^ LMT-55 peristaltic tubing (ID: 1.22 mm, OD: 2.92 mm, Saint-Gobain, Courbevoie, France) via PTFE 1/16” tubing with an inner diameter of 0.8 mm (BOLA™ PTFE tubing, Bohlender™ GmbH, Grünsfeld, Germany). The tubing for the buffers was connected to the microfluidic device via a microfluidic connector (4-way linear 4 mm connector; The Dolomite Centre Ltd., Royston, England). The tubing for the outlets was connected via flangeless fittings (Delrin^®^ 1/4-28 Flat-Bottom, IDEX Health & Science LLC, Middleboro, MA, USA) to 3D-printed 1/4″-28 threads.

**Figure A3:**
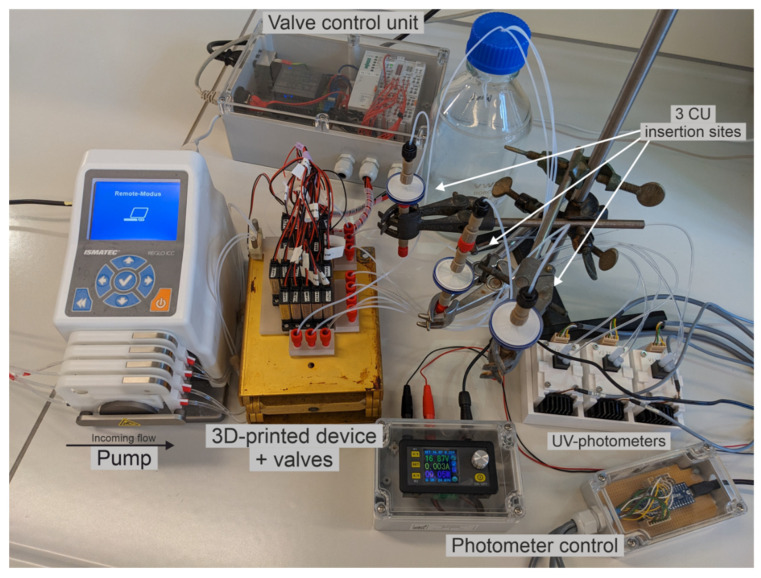
Photograph of the experimental setup with the 3D-printed PCCC device and its periphery for continuous chromatography.

Homogeneous mixing within the microfluidic device was achieved by merging the wash and elution buffer channels into an integrated microfluidic HC-mixer [32]. In order to prevent backflow, integrated microfluidic ball-check valves were used [26]. Twenty magnetic 3/2 solenoid valves (Whisper valve type 6724, Christian Bürkert GmbH & Co. KG, Ingelfingen, Germany) were mounted on the device to control the flow direction. A miniaturized self-constructed UV-photometer, equipped with a precision flow-through cell (optical path length 2 mm, volume 124 µL, Hellma GmbH & Co. KG, Mülheim, Germany), was also set in-line after each CU. A fraction collector (BioFrac™ Fraction Collector, BioRad, CA, USA) with a fraction size of 1 mL was then added to save selected fractions for further purification and analysis. A photograph of the experimental setup is included in Appendix A (Figure A3).

### 2.3. PCCC Process and System Control

**Figure A4:**
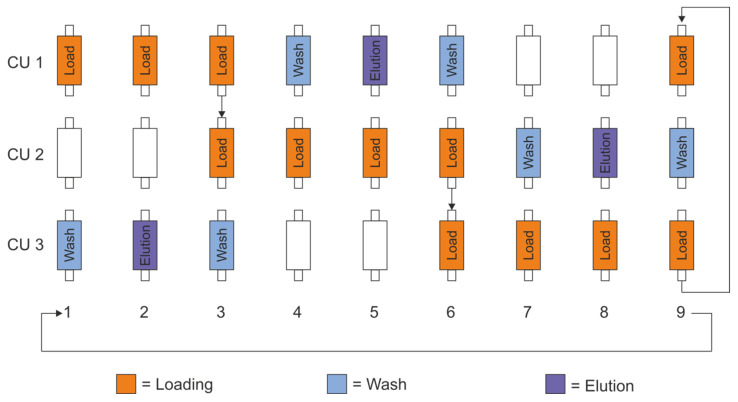
General steps of loading, washing, and elution in an example PCCC process.

In PCCC, three CUs are sufficient to run the purification continuously and thereby prevent product loss. The interconnection of two CUs during the loading process is the critical feature, and it was performed as follows: The first CU was loaded until its dynamic binding capacity was slightly exceeded. Then, the second CU was connected by switching valves so that some of the product contained in the feed stream bound at the remaining binding sides of the first CU, while the residual product was directed with the feed to the second CU. These two CUs remained connected until predefined switching conditions were reached. This principle was transferred to the other CUs, in order to achieve a cycling mode described in detail in Appendix A (Figure A4). A time-controlled approach was used to execute the steps of this PCCC process. A Python script for pump and valve control was created, and the time points for switching to the next PCCC step were entered. These time points were calculated via product breakthrough curves and were set for 10% and 50% of the dynamic binding capacity (DBC), respectively. The value of 10% was chosen in reference to previously published systems [22,23] that served as the initial model, whereas 50% was chosen to demonstrate procedure steps in quick succession.

### 2.4. Determination of Dynamic Binding Capacity

Dynamic binding capacity (DBC) was determined by reference to the generation of product breakthrough curves for each different type of CU. The feeding mAb solution was diluted to 1 mg∙mL^−1^, and its maximal absorbance was tested; afterwards, columns or membrane adsorbers were connected and loaded with a flow rate of 1 mL∙min^−1^ or 5 mL∙min^−1^, respectively, until a plateau was detected which matched the maximal absorbance measured earlier. The absorbance value of the plateau was set as the upper limit to 100% of product breakthrough, while the lower limit was set at absorbance values of HCP plateaus, or, if not applicable, to zero. All values were verified via HPLC.

### 2.5. Fabrication of UV-Photometers

**Figure A5:**
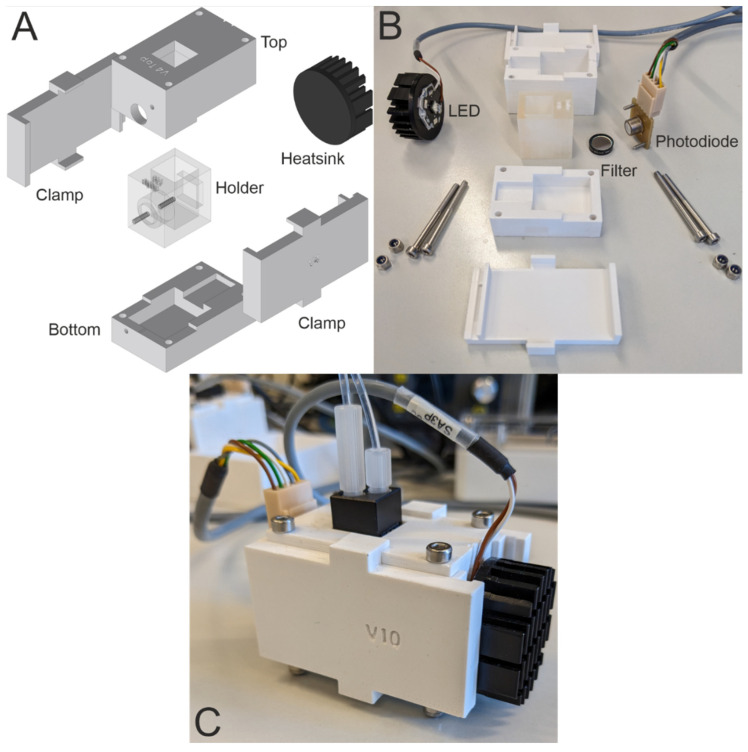
Overview of the self-constructed UV-photometer. The case holds the LED, cuvette holder and cuvette as well as the bandpass filter and photodiode. (**A**) An exploded view of the CAD drawings of the 3D-printed UV-photometer where the cuvette holder also contains a slot for the bandpass filter. The top and bottom parts enclose the cuvette holder, and the clamps fix the LED with its heat sink on one side and the photodiode on the other side. (**B**) All required parts for assembling the photometer. The screws and nuts for the top and bottom ensure that the photometer is shielded from ambient light. (**C**) The fully assembled UV-photometer.

**Figure A6:**
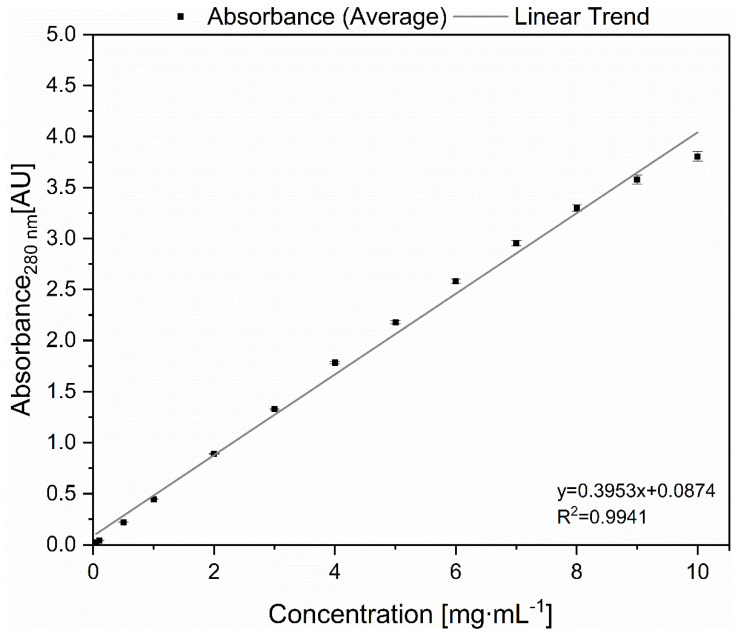
Calibration curve from the mean values of the three UV photometers with lysozyme solutions of different concentrations. Triplicates were used for each concentration.

To further miniaturize the system, the use of commercially available UV-spectrophotometers is not recommended, since these are bulky and have high acquisition costs. In addition, they complicate the extension and modification and, overall, the process integration. Therefore, photometers were customized for use with the PCCC. The case of these photometers was fabricated using 3D printing. The central element of the case is a holder for cuvettes. It is enclosed by a top and bottom part and fastened by two clamps. These clamps further secure a UV LED (UVR280-SA3P, Roithner Lasertechnik GmbH, Vienna, Austria) with an attached aluminum heat sink (ICK LED R 32 × 14 G, Fischer Elektronik GmbH & Co. KG, Lüdenscheid, Germany), in order to prevent overheating. Furthermore, a bandpass filter (280 nm CWL, 10 nm FWHM, Edmund Optics Inc., Barrington, NJ, USA) and a corresponding photodiode for the UV range (TOCON_B4, sglux GmbH, Berlin, Germany) were installed. CAD models for the case were designed using SolidWorks 2021. The holder was fabricated using the high-resolution 3D printer ProJet^®^ MJP 2500 Plus, as described above. All other parts of the case were 3D-printed using an Original Prusa i3 MK3S+ (Prusa Research a. s., Prague, Czech Republic) printer. The parts were printed with a layer height of 0.15 mm. Polylactic acid (DAS FILAMENT, Emskirchen, Germany) with a diameter of 1.75 mm was used as the filament for printing. The individual components and the assembly are shown in Appendix A in Figure A5. The customized photometers were tested in detail using different lysozyme concentrations. A calibration curve in the range from 0.005–10 g L^−1^ was obtained with a very high coefficient of determination (R^2^ = 0.9941). The limit of detection (LoD) of 0.0015 g L^−1^ was calculated using the standard deviation of the lowest concentration and the slope of the calibration curve. For the calibration curve, see Figure A6 in Appendix A.

### 2.6. Purification of mAb

In this study, an IgG1-mAb, produced in a fed-batch mode in CHO cells and cultivated in a 50 L stir-tank reactor in a serum-free medium, was used. Product characteristics included a molecular weight of 148 kDa and a pI (isoelectric point) of 8.25. After 12 days of cultivation, harvest was carried out and the mAb containing CHO supernatant was obtained via centrifugation. First, the supernatant was clarified of any larger particles via filter paper and vacuum pump featuring a pore size of 0.6 μm (BF, MACHEREY-NAGEL GmbH & Co. KG, Düren, Germany), followed by using a filter unit featuring a pore size of 0.2 µm (Sarstedt, Nümbrecht, Germany). For the chromatographic capture, Protein A affinity columns (HiTrap Protein A, 1 mL, Cytiva, Marlborough, MA, USA) were used in bind-and-elute mode. Equilibration was performed with 20 mM sodium phosphate and 150 mM NaCl buffer (pH 7.5). Columns were eluted with 100 mM citrate and 150 mM NaCl solution (pH 3.5). Cleaning-in-place for batch applications was carried out with 100 mM Tris-HCl, 500 mM NaCl (pH 8.5), and a contact time of 15 min maximum, followed by a regenerative wash. Fractions of 1 mL were taken and pooled if the mAb concentration exceeded 2 mg∙mL^−1^ and a purity of at least 70%. Prior to any further use, the solution was also filtered with a filter unit (0.2 µm).

The consecutive polishing step was performed via cation exchange chromatography using membrane adsorbers (Sartobind^®^ S15, Sartorius Stedim Biotech, Göttingen, Germany). Equilibration and washing buffer were 10 mM potassium phosphate, pH 6.7, while 1 M NaCl was added to the buffer for product elution. All chemicals were purchased by Carl Roth, Karlsruhe, Germany.

Based on the switching conditions mentioned in Section 2.3, the first CU was loaded exclusively until switching point 1 (17 min and 2 min for Protein A and CEX, respectively), where two units were then connected until the conditions for switching point 2 were fulfilled. Breakthrough curves were created for each application, and the resulting switching points are listed in Table 1. Switching point 1 was set to 10% product breakthrough to ensure comparability with the previously developed non-miniaturized in-house PCCC-system [33]. Switching point 2 was set to 50% of product breakthrough to limit the contact time of nearly product-free load and still demonstrate the successful implementation of the PCCC system.

### 2.7. Analytics via HPLC

Antibody samples were analyzed using high-performance liquid chromatography (HPLC) via a Yarra 3 µm SEC-3000 column (Phenomenex, Torrance, CA, USA) and 100 mM NaHPO_4_, 100 mM Na_2_SO_4_ mobile phase with a pH of 6.6, as described by Brämer et al. [33]. Samples of each elution fraction were tested and diluted with mobile phase buffer to a final product solution of 300 µg·mL^−1^. An amount of 5 µL of the sample solution was injected and run with a flow rate of 1 mL·min^−1^ for 20 min. The results were used in the calculations of mAb concentration and purity. Calculations of recovery and buffer consumption were made in reference to [34].

## 3. Results and Discussion

### 3.1. PCCC Design and Experimental Setup

**Figure 1:**
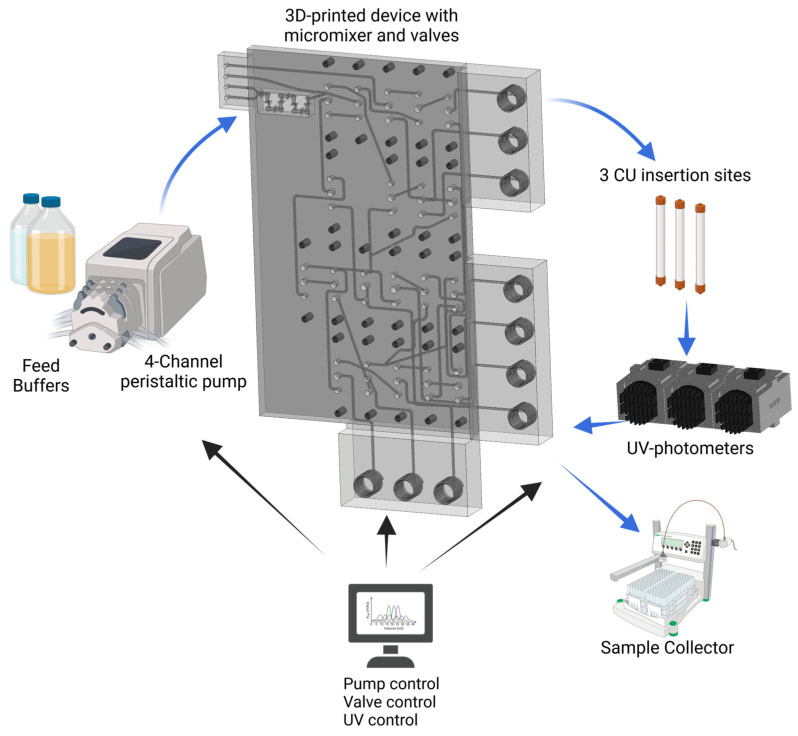
Schematic of the experimental setup of the 3D-printed PCCC system for continuous chromatography—including peripherals such as buffer reservoirs, peristaltic pump, fraction collector, three chromatographic units (CU), and UV-photometers. This system, including the pump, valves, and UV-photometers, is controlled and monitored via Python.

The miniaturized PCCC system was modeled after a three-unit PCCC system established by Brämer et al. [22]. In contrast to conventional PCCC systems, however, in this work a 3D-printed microfluidic device was used to replace most of the tubing with channels, thereby reducing the size of the overall system (while still allowing for the addition of required peripherals). Figure 1 depicts a schematic of the miniaturized PCCC system and its periphery for a general continuous chromatography setup.

In this experimental setup, the sample, wash buffer, elution buffer, and cleaning-in-place (CIP) buffer were introduced into the miniaturized 3D-printed system using a peristaltic pump. The wash and elution buffer channels conjoined within the device and led to an integrated mixing unit that enables homogenous mixing of the buffer solutions. Moreover, the device features three CUs with their own respective miniaturized and customized UV-photometer for protein quantification, as well as a waste outlet (also see Figure A1, Figure A2 and Figure A3 for circuit diagram, CAD drawing and photograph).

In order to connect the various internal channels and to facilitate the continuous PCCC process, 20 magnetic 3/2 solenoid valves were used to guide sample and buffer solutions through the device. First, one of the CUs was loaded until the dynamic binding capacity (DBC) was just exceeded, at which point the valves were then switched to connect the second CU. The residual and unbound product in the feed stream was then forwarded to the second CU. These two CUs remained connected until predefined switching conditions were reached. Subsequently, the first CU was regenerated by elution into the fraction collector and washing steps, and the feed stream was forwarded from the second to the third CU after exceeding the DBC. When the third CU exceeded its DBC, the first CU was sufficiently regenerated already and the whole PCCC process could thus be employed continuously. Please also refer to Appendix A and Figure A4 for a more detailed description of the PCCC process.

**Figure 2:**
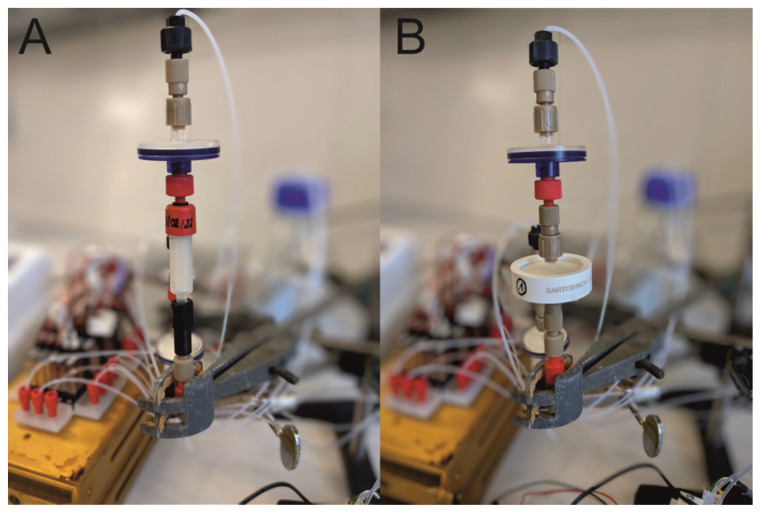
Installation of (**A**) a small preparative chromatography column and (**B**) a membrane adsorber into the PCCC system.

The system and its different unit operations (e.g., pump, valves, and UV-photometers) is controlled by a Python script and could thus be automated. Furthermore, this setup can be readily modified and expanded (e.g., with different filter units and additional sensors for process monitoring). In addition, the miniaturization of the system simplifies integration into different processes. For instance, small preparative columns or membrane adsorbers can be easily exchanged (see Figure 2). This feature proved to be of great importance, as it allowed for a change from the initial Protein A affinity-based capture step (Figure 2A; column) to a cation exchange (CEX) chromatography protocol (Figure 2B, membrane adsorber). This latter step is a polishing process which effectively removes any remaining impurities, such as aggregates and HCPs, from the acquired antibody solution. The total system has a dead volume of approximately 6.79 mL, with the 3D-printed device accounting for only 0.62 mL.

In the following, the use of the 3D-printed PCCC system for the initial Protein A-based mAb capture and a membrane adsorber-based CEX polishing step is demonstrated.

### 3.2. Protein A Affinity Chromatography

**Figure 3:**
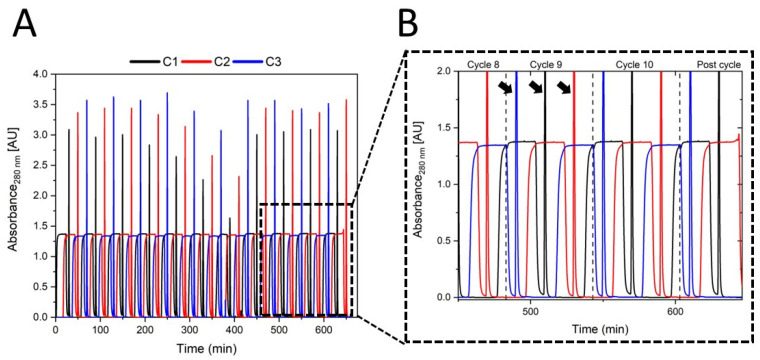
(**A**) Chromatogram of continuous Protein A-based purification performed with three columns numbered C1–C3. The run was performed over 650 min, including 10 cycles with three elution steps each. Columns of 1 mL were used. Loading plateaus were consistent in height and at regular intervals throughout the process. (**B**) Zoomed-in section from 450 min to 650 min and cut-off at 2.0 AU. Arrowheads exemplarily indicate elution peaks for all CUs in cycle 9.

First, the miniaturized system was used with commercial Protein A columns for mAb capture from crude cell culture supernatant. Ten PCCC cycles were carried out—with each cycle consisting of loading, washing, and elution of each column one time, resulting in 30 elution steps plus two cleaning elutions at the end of the process. Using this setup and process, elutions were obtained approximately every 20 min—a substantial reduction from the 34 min typically required to complete a traditional batch purification with load (10% DBC), wash, elution, and regeneration time. The resulting chromatogram is shown in Figure 3A, and a zoom-in to highlight details is depicted in Figure 3B.

The continuous chromatogram shows breakthrough plateaus during loading and elution peaks. The loading was characterized by plateaus that reached a value of 1.3 AU and can be ascribed to host cell proteins that are present in the feed stream and do not bind to the Protein A ligand of the chromatography matrix. Loading times and interconnection times were determined via recorded dynamic binding curves, as shown in Table 1. Overall, a feed load of 60 mL∙h^−1^ was possible, as feed was pumped at every step of the cycle, and the flow rate was restricted to the maximal flow rate of the Protein A columns (1 mL∙min^−1^). The correspondingly expected product breakthrough was not detected; this might be ascribed to small variations of mAb concentration within the feed solution. Longer loading times or higher product concentration could be helpful in utilizing the column capacity even further. In contrast, the elution peaks are characterized by sharp peaks (exemplarily emphasized by arrows for cycle 9 in Figure 3B) that reached values of 3–3.5 AU at the beginning of the continuous chromatography process. During the process, it was noted that the peak height decreased—which can be explained by the blocking of the inserted filter units installed to protect the chromatography columns from large particles. The blocked filters led to an increase in backpressure and a visible but slight flow rate decrease at the outlets. The filters were replaced when the relevant CU position was under washing flow, and from minute 430 onward, the elution peak heights of earlier cycles were retained. Interestingly, the blocked filters did not have any visible impact on the height of the breakthrough plateaus during loading, which we attribute to their broader shape. All elution samples (time point of greatest peak height) were analyzed by HPLC-SEC, and an average purity of 95.46% with concentrations up to 13 mg·mL^−1^ was observed. This means that the obtained purity levels match traditional batch standards, even though the product concentration exceeded the observed value of 8 mg·mL^−1^ for our in-house established batch process [18]. As no HCPs were present in the analyzed samples, minor reductions in the purity were exclusively traced back to the detected aggregate content—an issue that also occurs in similar processes [35]. Compared to an ideal batch run, 25% more product was recovered due to the interconnection of several columns, and mAb product loss by extensive overloading was also prevented. Indeed, compared to other continuous chromatography systems (such as a conventional PCCC), the amounts of mAb per cycle was observed to remain the same even though dead volume and buffer consumption were both significantly reduced [33]. The staggered manner allowed the requirement of just 1.57 mL∙mg^−1^ buffer per mg purified mAb, which was 30% less compared to the batch process [36]. It should be noted that the commercial columns used in this experiment were chosen because of their relatively low cost and short loading times to demonstrate the functionality of this proof-of-concept system itself. The use of higher-quality columns could, of course, further improve the system, but using such new units would require adaptation and performance re-evaluation [37].

### 3.3. CEX for Product Polishing

The initial capture step is usually followed by polishing steps, which aim to remove the remaining impurities from the acquired antibody solution. A typical method for this polishing step is CEX, which is very useful for the removal of aggregates and HCPs [18]. In the presented PCCC system, the chromatography columns from the previous step are easily replaced by MAs for CEX (also refer to Figure 2).

**Figure 4:**
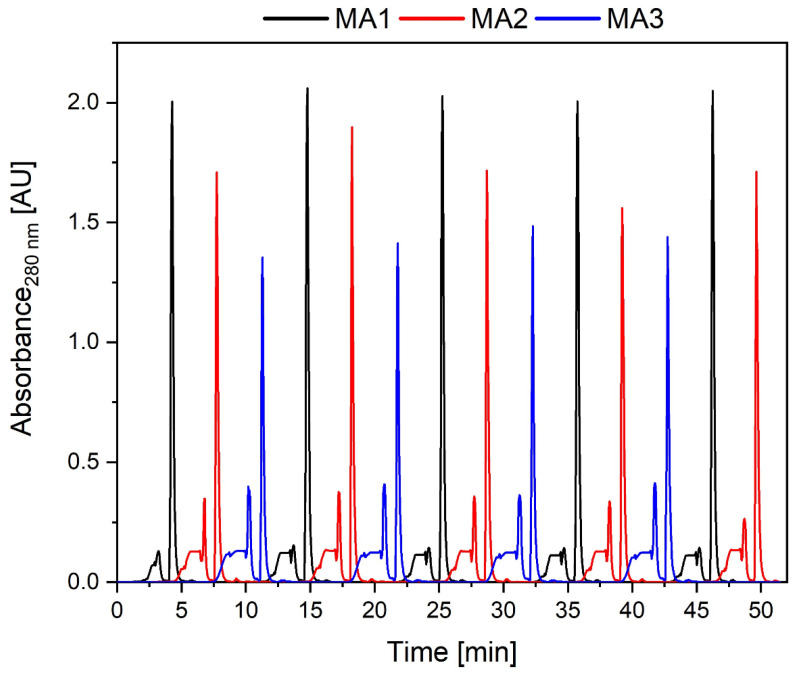
Chromatogram of a continuous membrane adsorber-based CEX run using three membrane adsorbers numbered MA1–MA3. A total of 4 cycles (55 min) and a cleansing post cycle using membrane adsorbers with a volume of 0.41 mL were performed. Small regular plateaus with peaks at the end emerge during loading.

These MAs have higher throughputs and shorter cycle and residence times compared to columns. They, therefore, allow for doubling the number of elution steps per hour vis-à-vis polishing the mAb. Furthermore, up- and down-scaling is easier when using MAs, and they are also disposable (although they have lower capacities than chromatography columns) [21]. The continuous CEX chromatography process for mAbs polishing using these MAs is shown in Figure 4. Breakthrough plateaus (before the higher elutions peaks) can once again be attributed to impurities that did not bind to the MAs or starting overload. Small spikes at the end of these plateaus are ascribed to switching the valves (from loading to washing) at high flow rates and introducing small air bubbles that cause such characteristic “down and up” signals. This effect could not be avoided due to the PCCC programming and the time-controlled setup.

On average, 258.75 mL∙h^−1^ feed could be applied to the system. The flow rate was restricted by the pump rates and the backpressure of the system, although theoretically, the applied MAs can be used at higher flow rates. The differences in peak intensities between the three membrane adsorbers can be attributed to the use of three UV photometers that are structurally the same but may show variations due to minor differences between the LEDs and photodiodes. Moreover, minor differences in the protein binding capacity of the MAs (although produced in the same batch) might contribute to peak height differences. This effect was especially noticeable at high intensities outside the linear range. The system was fully functional, and there was no significant height change in the elution peaks with respect to each individual adsorber during the process. Moreover, the purification was reliable—obtaining an average product purity rate of 97.2% and a mAb concentration of 7.43 mg∙mL^−1^ with a reduced aggregate content. The mAb concentration was lower than for the capture step as the MAs have a lower dynamic binding capacity. However, the maximal recovery per MA was still increased by 16% due to overloading when compared to direct batch load. Because of the staggered approach of the PCCC, the buffer consumption was reduced to the same extent as for the capture step. More elutions per hour were possible, and thus, the space–time yield increased. Further improvements could potentially be achieved by optimizing loading times or using UV-based process control, allowing cycle step adjustments for each CU individually.

## 4. Conclusions

The presented system demonstrates the successful miniaturization of a PCCC platform for continuous capture (Protein A affinity) and polishing (CEX) chromatography processes of a mAb using 3D printing technology. By leveraging the benefits offered by this system, dead volume was reduced by more than 55% when compared to the 3MA-PCCC system reported by Brämer et al., significantly reducing buffer consumption by up to 30% [33]. In addition, the space–time yield was also improved compared to batch processes by increasing the number of elution steps per hour (which was enabled by the parallelization of the chromatography steps). Due to the overloading—a characteristic of the PCCC approach—batch recovery was exceeded by up to 25% and 16%. Further optimization could be achieved by improving the switching conditions (e.g., UV-controlled switching). Thanks to significant reductions in size and weight compared with systems already available on the market, e.g., ÄKTA PCC by Cytiva, it bears noting that this novel 3D-printed system is also essentially portable, which could help to facilitate its practical application in different laboratory environments [38,39]. With overall costs of ~15,000 EUR, the entire miniaturized PCCC system offers a great chance as a cost-effective alternative, especially considering that there is a research demand to simplify continuous processes for future utilization in biotechnological production [40]. Compared to commercially available systems, the developed system is in the range of the most basic batch systems [41] but much cheaper than more sophisticated chromatography systems. Importantly, the 3D-printed miniaturized device itself accounts for only 55 EUR. Thus, replacement with adapted 3D-printed devices for changing experimental requirements is feasible and not too expensive.

This miniaturized PCCC system is readily customizable, thanks to 3D printing. It is, therefore, feasible to extend the system and integrate sensors (i.e., measuring conductivity, pressure, etc.) to the periphery of the device, which allows for further enhanced process control. To facilitate the execution of several PCCC cycles and improve process control, as a next step, we envision establishing a UV-controlled setup. With such UV control, the user would be able to adjust loading times precisely to the properties of the feed material, compensating for minor variations in the process and exploiting the advantages of continuous chromatography even more.

Generally, this system could be used for any product purified via bind-and-elute separation techniques. As such, it could be leveraged to accomplish a multitude of possible purification tasks, ranging from recombinant proteins to oligonucleotides. We envision attaching the system directly to perfusion cell culture in future applications, which would be particularly well-suited for capturing unstable and sensitive products that would otherwise risk suffering damage if not directly purified and recovered quickly and constantly. Finally, connecting two miniaturized customized systems for product capture and polishing, together with perfusion cell culture, could also constitute a major step towards realizing a fully integrated, continuous, and automated downstream process.

## Figures and Tables

**Table 1:** Overview of switching conditions for time-controlled mAb purification process and chosen chromatography units.

Method	Direct Load	Indirect Load	Flow Rate	10% DBC	50% DBC
Protein A column	17 min	5 min	1 mL∙min^−1^	17 mg	21.8 mg
CEX MA	2 min	0.5 min	5 mL∙min^−1^	8.78 mg	10.89 mg

## Data Availability

Data are available upon reasonable request from the corresponding author.

## Appendix A

The miniaturized PCCC system (see Figure A1) requires four inlets—one for the sample, one for the wash buffer, one for the elution buffer, and one for the cleaning-in-place (CIP) buffer. The channels for the wash and elution buffer are merged within the device and incorporate an integrated mixing unit to enable homogenous mixing of the buffer solutions. In order to connect the various internal channels of the device, 20 magnetic 3/2 solenoid valves are used. The device features ten outlets to connect the device to its periphery: three for each of the three CUs, including UV photometers (inlet, outlet, and waste required, respectively), and a tenth outlet for elution (fraction collector). The channels within the device are rectangular, have a width and height of 0.5 mm, a total channel length of approx. 2250 mm and an internal volume of approx. 0.62 mL. A schematic of the internal and external circuitry is provided in Figure A2.

The first CU is loaded until the dynamic binding capacity is slightly exceeded and the breakthrough is approaching. Thus, in the next step, the feed is directly loaded onto the second CU until the breakthrough point is approaching again, and a connection to the third CU is needed in order to avoid product loss; meanwhile, the first CU is simultaneously being washed, eluted, and re-equilibrated. After matching the switching conditions, the third CU is loaded directly, and the freshly regenerated first CU is loaded with the breakthrough while the second CU is washed, eluted, and re-equilibrated. A whole cycle is completed when each CU has been directly loaded one time. The best utilization of the system is given when the loading time equals the time for regeneration. The general order of steps is shown in Figure A4.
